# IκBNS expression in B cells is dispensable for IgG responses to T cell-dependent antigens

**DOI:** 10.3389/fimmu.2022.1000755

**Published:** 2022-10-21

**Authors:** Sharesta Khoenkhoen, Monika Ádori, Darío Solís-Sayago, Juliette Soulier, Jamie Russell, Bruce Beutler, Gabriel K. Pedersen, Gunilla B. Karlsson Hedestam

**Affiliations:** ^1^ Department of Microbiology, Tumor and Cell Biology, Karolinska Institutet, Stockholm, Sweden; ^2^ Center for the Genetics of Host Defense, University of Texas (UT) Southwestern Medical Center, Dallas, TX, United States; ^3^ Center for Vaccine Research, Statens Serum Institut, Copenhagen, Denmark

**Keywords:** nfkbid, NF-κB, conditional, CD79a, B cell responses, IκBNS

## Abstract

Mice lacking the atypical inhibitory kappa B (IκB) protein, IκBNS, a regulator of the NF-κB pathway encoded by the *nfkbid* gene, display impaired antibody responses to both T cell-independent (TI) and T cell-dependent (TD) antigens. To better understand the basis of these defects, we crossed mice carrying floxed *nfkbid* alleles with mice expressing Cre under the transcriptional control of the *Cd79a* gene to create mice that lacked IκBNS expression only in B cells. Analyses of these conditional knock-out mice revealed intact CD4^+^ and CD8^+^ T cell populations, including preserved frequencies of FoxP3^+^ regulatory T cells, which are known to be reduced in IκBNS knock-out mice. Like IκBNS knock-out mice, mice with conditional IκBNS ablation in B cells displayed defective IgM responses to TI antigens and a severe reduction in peritoneal B-1a cells. However, in contrast to mice lacking IκBNS altogether, the conditional IκBNS knock-out mice responded well to TD antigens compared to the control mice, with potent IgG responses following immunization with the viral antigen, rSFV-βGal or the widely used hapten-protein model antigen, NP-CGG. Furthermore, B cell intrinsic IκBNS expression was dispensable for germinal center (GC) formation and T follicular helper cell responses to NP-CGG immunization. The results presented here suggest that the defect in antibody responses to TD antigens observed in IκBNS knock-out mice results from a B cell extrinsic defect.

## Introduction

B cells constitute an important arm of the adaptive immune system, by secreting antibodies to neutralize, contain or opsonize pathogens. B cell activation occurs either in a T cell-dependent (TD) manner to protein antigens, or in a T cell-independent (TI) manner, such as in response to Toll-like receptor (TLR) agonists (TI-1) or polysaccharides (TI-2). The latter contain repetitive structural determinants that activate B cells *via* B cell receptor (BCR) ligation. TI responses are not subjected to affinity maturation and only limited memory to this type of antigens has been described (1). In contrast, TD responses are associated with somatic hypermutation of rearranged antibody genes and selection of the B cells with high affinities to the cognate antigen, as well as with the formation of memory B cells and long-lived plasma cells (2). These hallmarks of the TD response require the germinal center reaction, a central component of the TD immune response (3).

Combined variable deficiency (CVID) is a disorder, which is manifested by hypogammaglobulinemia, rendering affected people susceptible to infections. CVID often involves mutations in genes important for development and/or function of B cells (4). Whilst there is a strong genetic component to CVID (5), more than 80% of CVID cases have no identified underlying mutations (4), suggesting that screening for genes not previously implicated in CVID could lead to identification of additional underlying genetic causes. Recent studies have identified a number of mutations in the NF-κB pathway as underlying genetic cause of CVID, e.g. in NF-κB1 (6, 7), NF-κB2 (8), and NEMO (9).

The NF-κB transcription factors, p50 (NF-κB1), p52 (NF-κB2), p65 (RelA), c-Rel and RelB, regulate transcription by binding to promoters of target genes. Inhibitors of κB (IκB) proteins, such as IκB-α, IκB-β and IκB-ϵ and the p50 precursor p105 sequester NF-κB transcription factors in the cytoplasm, thus rendering them inactive (10). BCR activation leads to classical NF-κB signaling, in turn targeting IκBs for proteasomal degradation and releasing the NF-κB1 p50/RelA or p50/c-Rel complexes for translocation to the nucleus (11–13). The NF-κB transcription factors may be further regulated by a class of atypical IκB proteins that include BCL-3, IκBζ, IκBNS and IκBη (14).

Our studies have focused on the IκB family member, IκBNS. The IκBNS-deficient *bumble* strain displays reduced B-1 and marginal zone B (MZB) cell numbers and impaired responses to TI and TD antigens (15–17). IκBNS haploinsufficient mice also display impaired TI-2 responses, despite having normal B-1 and MZB cell numbers (18), suggesting that IκBNS is not only required for the generation of these B cell subsets, but also for BCR signaling in mature B cells.

Here, we investigated the role of IκBNS in the response to TD antigens. Using the *bumble* mice, which lack IκBNS expression due to a point mutation in the *nfkbid* gene, we previously demonstrated a requirement for IκBNS in primary IgG responses to recombinant Semliki Forest virus expressing the heterologous antigen β-Galactosidase (rSFV-βGal) (15). We also observed delayed IgG responses to NP-CGG in adjuvant in *bumble* mice suggesting that even a strong TD antigen requires IκBNS for an intact B cell response (15). Others have demonstrated that IκBNS knock-out (KO) mice display delayed IgG responses to the TD antigen TNP-KLH and defective GC formation after sheep red blood cell immunization (17). The generation of T follicular helper (T_FH_) cells has been shown to be dependent on IκBNS expression, possibly through direct induction of *Bcl6*, *IL21*, and *Cxcr5* gene expression (19). Thus, the role of IκBNS in the response to TD antigens remains incompletely understood, including if B cell intrinsic IκBNS expression is required for B cell activation and GC formation.

To investigate the role of IκBNS in B cells during the primary immune responses to TD antigens, we generated mice with conditional deletion of the *nfkbid* gene, which encodes IκBNS, in B cells, named *nfkbid* ^B-^. Analyses of TD antibody responses in these mice suggested that IκBNS expression in B cells was dispensable for GC B cell formation and antigen-specific IgG production, whereas TI responses were defective.

## Materials and methods

### Mice

Mice were maintained at the animal research facilities at Karolinska Institutet. Studies were performed in accordance with institutionally approved protocols and Committee for Animal Ethics (Stockholms Norra Djurförsöksetiska nämnd) approval. The *Cd79a* ^Cre^ strain (20) was kindly provided by Michael Reth. The *nfkbid* ^tm1a^ strain was obtained from the EUCOMM repository. To generate *nfkbid* ^tm1c^ (*nfkbid* ^fl/fl^) mice, the *nfkbid* ^tm1a^ strain was first crossed to ROSA26^Fki^ mice to remove the *Frt*-flanked *lacZ* and *neo* reporter cassettes from the *nfkbid* alleles through Flp recombinase-mediated deletion. The *Cd79* ^Cre/+^ *nfkbid* ^fl/fl^ strain was generated by breeding *Cd79a* ^Cre/+^ and *nfkbid* ^fl/fl^ mice to obtain *Cd79a* ^Cre/+^ *nfkbid* ^fl/wt^ mice first, followed by a second cross between *Cd79a* ^Cre/+^ *nfkbid* ^fl/wt^ mice and *nfkbid* ^fl/fl^ mice, which was dependent on a chromosomal crossover event as both the *Cd79a* locus and the *nfkbid* locus are located on chromosome 7. The *Cd79a* ^Cre^ *nfkbid* ^fl/fl^ strain was then maintained through breeding on hemizygous *Cd79* ^Cre/+^ background and mice inheriting the *Cre* gene were identified by genotyping PCRs performed on ear biopsies. Mice were used at 6-12 weeks of age for experiments and euthanized using gradual carbon dioxide displacement up to 22% for approximately three minutes.

### Genotyping

Ear biopsies were incubated for 50-60 minutes in 75 µl solution containing 25 mM NaOH and 0.2 mM EDTA at 95°C, put on ice to reduce the temperature, after which 75 µl solution containing 40 mM Tris HCl (pH 5.5) was added (21). PCR reaction was prepared using DreamTaq Green PCR Master Mix (Thermo Fisher), 25 pmol of the *forward* and the *reverse* primer each, and 5 µl of template in a total reaction volume of 25 µl. PCR amplification of the *Cd79a-Cre* allele was performed using the *forward* and *reverse* primers 5’-CCCTGTGGATGCCACCTC-3’ and 5’-GTCCTGGCATCTGTCAGAG-3’ (22) resulting in a 450-bp amplified DNA fragment. PCR amplification of the *Cd79a* wildtype allele was performed using the *forward* and *reverse* primers 5’-GGCTCTGACCCATCTGTCTC-3’ and 5’-CCTTGCGAGGTCAGGGAGCC-3’ (20), resulting in a 477-bp amplified DNA fragment. PCR amplification of *nfkbid* wildtype and *nfkbid* floxed alleles was performed using the *forward* and *reverse* primers 5’- TCCATGAGGTAGGGATGGAGAGTA-3’ and 5’- GAAAGAGGATCTCACTGTGAAGTC-3’, resulting in 235-bp and 452-bp amplified DNA fragments specific for the wildtype and floxed alleles, respectively. The following conditions were used for amplification of DNA fragments: denaturation at 95°C for 2 minutes, PCR amplification at 95°C for 30 seconds, 56°C for 30 seconds, and 72°C for 60 seconds for 29 cycli, followed by 7 minutes at 72°C.

### Cell preparation

Splenocytes, lymph nodes, and thymi were prepared as single cell suspensions using 70 μm cell strainers in RPMI 1640 (HyClone) supplemented with 10% fetal bovine serum (FBS) (HyClone), penicillin (100 IU)-streptomycin (100 μg mL^-1^) (Sigma), β-mercaptoethanol (0.05 mM) (Gibco) (complete RPMI). Splenocytes and thymocytes were washed once in Ca^2+^- and Mg^2+^-free PBS (Sigma), treated with 1 ml of red blood cell lysis buffer for 1 minute, and washed twice in PBS before further processing. Peritoneal cells were obtained by injecting and withdrawing 8-10 ml of PBS into the peritoneal cavity. To obtain lymphocytes from blood for flow cytometry, 2-3 drops of blood collected in 1.5 mL Eppendorf tubes containing 20 µL EDTA. Blood was treated twice with 2 ml red blood cell lysis buffer for 4 to 5 minutes and washed in PBS before further processing. For ELISA, blood was allowed to coagulate at room temperature for one hour, spun down at 6000 rpm for 6 minutes, and serum was collected and stored at -20°C.

### Immunization

Mice were immunized intraperitoneally (i.p.) with 50 μg NP(40)-Ficoll (Biosearch Technologies) or 2x10^6^ IU rSFV-βGal in a total volume of 100 μl PBS. NP(49)-CGG (Biosearch Technologies) was diluted in PBS and mixed 1:1 in AddaVax (Invitrogen). Mice were immunized i.p. with 5 µg or subcutaneously (s.c.) with 25 or 2.5 µg NP(49)-CGG on both flank regions in a volume of 100 μl PBS.

### Real-time PCR

RNA was isolated from 3x10^6^ B cells using Trizol (Invitrogen) followed by DNase-treatment using TURBO DNA-free kit (Thermo Fischer) according to the manufacturer’s instructions. RNA concentration was measured on Qubit (Thermo Fischer). cDNA synthesis was performed with 100 ng of RNA using SuperScript IV (Invitrogen) according to the manufacturer’s instructions. Real-time PCR was prepared with 1 μL of cDNA and 1 μM of the *forward* and *reverse* primer in RT^2^ SYBR Green Master Mix (Bio-Rad) in a total volume of 10 μL. For IκBNS mRNA expression level, *forward* and *reverse* primer sequences used for amplification were 5’-CTCCATCTGTGAATGAGGCAGAGC-3’ and 5’-AGATCCACTTGAATGCCGGACTTAAAC-3’, respectively (15). Assays were performed in 384-well plates on Bio-Rad CFX384 thermal cycler under the following conditions: denaturation at 95°C for 2 minutes, PCR amplification at 95°C for 5 seconds and 60°C for 20 seconds for 45 cycles, followed by melt-curve analysis of 0.5°C increments per 5 seconds from 65°C to 95°C.

### ELISA

ELISA plates (Nunc) were coated with 2 µg/ml β-Galactosidase (Sigma) or 500 ng/well of NP(30) conjugated with BSA (Biosearch Technologies). To detect IgM, IgG, IgG1, IgG2b, IgG2c, IgG3 or IgA plates were coated with unconjugated goat anti-mouse IgM, IgG or IgA (Southern Biotech). After incubation overnight (4°C), washing with PBS + 0.05% Tween20 and blocking for 1 h with PBS containing 2% dry milk (blocking buffer), 5 μl of serum was added in a total volume of 150 μl, followed by 3-fold serial dilutions in blocking buffer and incubated for 2 h at room temperature (RT). Plates were washed six times, and primary antibodies, biotinylated goat anti-mouse IgM, or goat anti-mouse IgG (both from Mabtech AB), biotinylated goat anti-mouse IgA (BD Pharmingen), or HRP-coupled anti-IgG1, anti-IgG2b, anti-IgGc or anti-IgG3 (Southern Biotech) were added in 100 μl PBS/well followed by incubation for 1.5 h, at RT. Streptavidin-HRP was added to biotinylated antibodies in 100 μl PBS/well after washing six times and incubated for 1 h, at RT. The assay was developed with TMB substrate (KPL), the reaction was stopped with 1 M H_2_SO_4_, and the OD was read at 450 nm using an Asys Expert 96 ELISA reader (Biochrom Ltd.).

### ELISpot

Plates were activated with 100 µl 70% ethanol, washed three times in PBS, and coated overnight at 4°C with 500 ng/well of NP(30) conjugated with BSA (Biosearch Technologies). Plates were washed three times with PBS and blocked with 200 µl complete medium per well for 1 h at 37°C. Splenocytes were added at 5x10^5^, 2.5x10^5^, 1x10^5^ or 5x10^4^ cells in 100 µl complete medium per well in triplicate and incubated for 12 h at 37°C. Plates were washed six times in PBS + 2% Tween20 before addition of biotin-coupled anti-mouse IgG (Mabtech AB), followed by 2 h incubation at room temperature. Plates were washed six times in PBS + 2% Tween20 and 100 μl/well streptavidin-alkaline phosphatase was added. After 45 minutes incubation on RT, plates were washed and developed with 100 µl/well 5-bromo-4-chloro-3-indolyl phosphate (BCIP)/NBT plus substrate (Mabtech AB) for 10-15 minutes. The reaction was stopped by excessively washing the plates with tap water. Plates were left to dry overnight before analysis on a CTL Immunospot.

### Flow cytometry

To block nonspecific binding to Fc receptors, cells were incubated with anti-CD16/32 antibody (BD), and then stained with different panels of fluorochrome-conjugated monoclonal antibodies (**Supplementary Table 1**) in PBS/2% FBS. Samples were run using a BD FACS Celesta flow cytometer and data were analyzed in FlowJo v10.6.1 (Treestar). Cell populations are pre-gated on singlet (FSC-W *vs*. FSC-A) and lymphocyte (SSC-A *vs*. FSC-A) subsets prior to further gating as indicated in figure legends.

### Statistics

Differences between groups were analyzed by a Mann-Whitney test (GraphPad Prism v8). Statistical significance is indicated with * for *P* ≤0.05, ** for *P* ≤0.01, *** for *P* ≤0.001, **** for *P* ≤0.0001, and ns for not significant.

## Results

### Generation of conditional IκBNS knock-out mice

**Figure 1:**
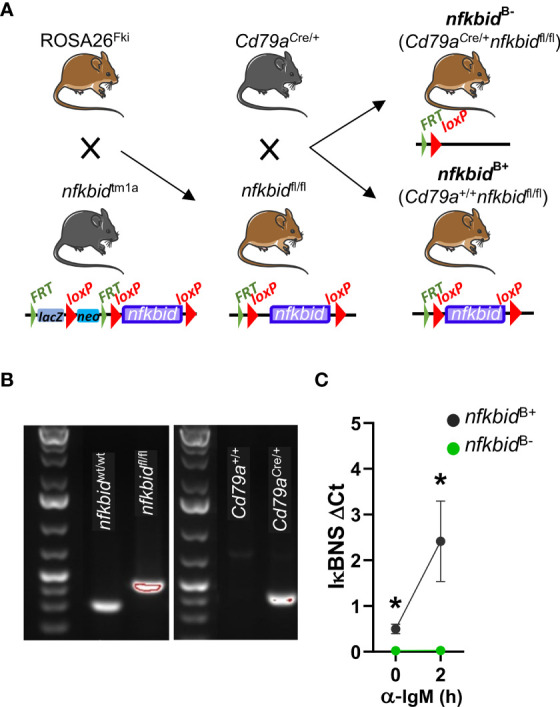
Generation and genotyping of B cell-specific IκBNS knock-out mice. **(A)** Schematic overview of mouse strain crosses and targeted modification of the *nfkbid* locus. The *Frt*-flanked *lacZ* and *neo* cassettes were removed from the *nfkbid* ^tm1a^ allele through Flp recombinase-mediated excision by crossing *nfkbid* ^tm1a^ mice to ROSA26^Fki^ mice. This created *nfkbid* alleles in which the coding exons 4 to 8 are flanked by *loxP* sequences in the offspring *nfkbid* ^fl/fl^ mice. Next, these mice were crossed to *Cd79a* ^Cre/+^ mice in which the Cre recombinase is expressed under the B cell-specific promotor of the Igα chain, creating *Cd79a* ^Cre/+^ *nfkbid* ^fl/fl^ mice in which exons 4 to 8 of the *nfkbid* alleles were deleted resulting in loss of *nfkbid* expression in the B cell compartment. **(B)** Genotypes of mice were determined by PCR analysis of ear samples. Representative gel images show wt *nfkbid* allele (*nfkbid* ^wt/wt^) and floxed *nfkbid* allele (*nfkbid^fl^* ^/fl^) (left), the cre-mb1 allele (present in *Cd79a* ^Cre/+^ and absent in *Cd79a^+^* ^/+^) (right), as indicated. **(C)** *nfkbid* mRNA expression relative to *PolR2A* in splenic B cells at steady-state and after stimulation for 2 h with anti-IgM. Means ± SD are plotted. Data is from one experiment with 3 mice per group. Mouse pictures were created with https://BioRender.com. *P ≤0.05.

To investigate the role of IκBNS in antibody responses against TD antigens, we generated a mouse strain in which *nfkbid* was selectively deleted in the B cell compartment. We first removed the *lacZ* and *neo* cassettes from the *nfkbid* ^tm1a^ strain through Flp-mediated recombination by crossing to the ROSA26^Fki^ strain. The resulting *nfkbid* ^fl/fl^ mice were crossed to the *Cd79a* ^Cre/+^ strain to mediate deletion of the loxP-flanked exons of *nfkbid* at the transition from the common lymphoid progenitor to the pro-B cell stage in developing B cells (20). These mice were intercrossed to obtain littermate cohorts of *Cd79a* ^Cre/+^ *nfkbid* ^fl/fl^ (experimental) and *Cd79a* ^+/+^ *nfkbid* ^fl/fl^ (control) mice, hereafter referred to as *nfkbid* ^B-^ and *nfkbid* ^B+^ mice, respectively (**Figure 1A**). Prior to each experiment, the genotype of mice was confirmed by PCR analyses (**Figure 1B**). To verify the deletion of the *nfkbid* alleles, we also evaluated *nfkbid* mRNA levels in isolated splenic B cells after two hours of α-IgM stimulation. We did not detect any *nfkbid* mRNA expression at steady state or after stimulation in B cells from *nfkbid* ^B-^ mice (**Figure 1C**).

**Figure 2:**
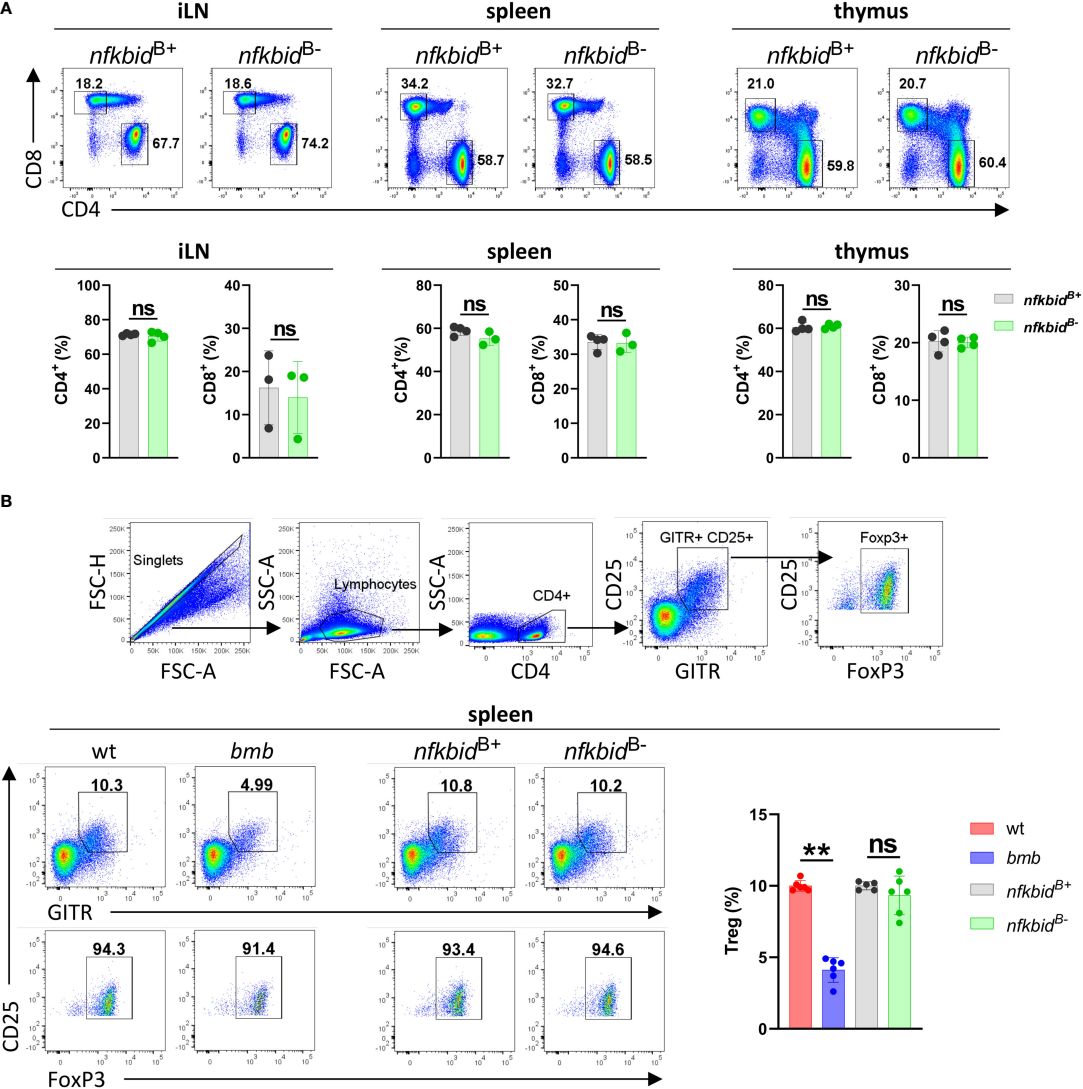
Intact T cell compartments in the *nfkbid* ^B-^ conditional knock-out mice. **(A)** Representative flow cytometry plots showing gating strategy for CD4^+^ and CD8^+^ T cells in inguinal lymph nodes (iLN), spleen and thymus (upper row). Cell populations shown are pre-gated as B220^-^ CD3^+^. Frequencies of CD4^+^ and CD8^+^ T cells (lower row). Data is representative of three experiments with 3-5 mice per group. **(B)** Flow cytometry plots showing gating strategy for CD4^+^ CD25^+^ GITR^+^ FoxP3^+^ T_regs_ (upper FACS panels). Representative flow cytometry plots show T_regs_ (lower row, left FACS panels) and their frequencies within the CD4^+^ population (right graph bars) in spleen from wt, *bumble* (*bmb*), *nfkbid* ^B+^, and *nfkbid* ^B-^ mice. Data is representative of three experiments with 5-6 mice per group. Numbers adjacent to gates indicate cell frequencies in the parent population. Bars and error bars represent mean ± SD. **for *P* ≤0.01, ns, not significant

Next, we assessed possible off-target deletion of *nfkbid* in T cells. We analyzed the CD4^+^, CD8^+^ and the T_reg_ compartment in the spleen, inguinal lymph node (iLN), and thymus. Generation of intact T_reg_ cells has been shown to be dependent on IκBNS-induced expression of FoxP3 (21). In IκBNS-deficient *bumble* mice, the frequency of CD25^+^ FoxP3^+^ T_reg_ cells were severely decreased compared to in wt control mice. In contrast, we found the frequencies of the examined T cell populations to be similar in the *nfkbid* ^B-^ mice compared to the *nfkbid* ^B+^ mice (**Figures 2A, B** and **Supplementary Figure 1**), suggesting that no off-target deletion of *nfkbid* occurs in the T cell compartment of *nfkbid* ^B-^ mice.

### B-1 cell development and TI antigen responses require IκBNS expression in B cells

**Figure 3:**
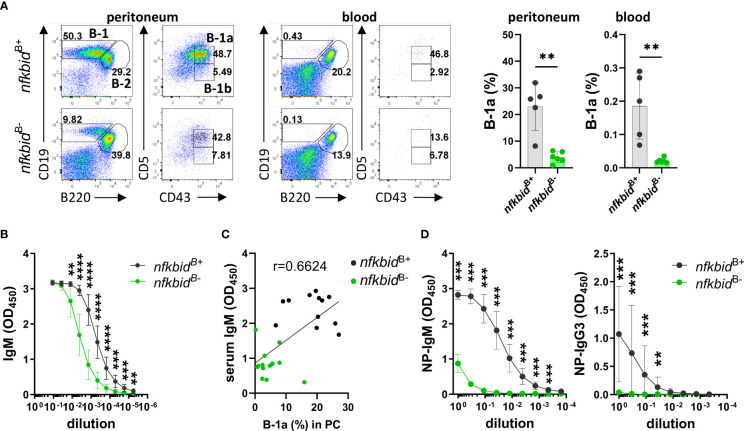
Reduced B-1a B cell compartment and TI immune response in IκBNS conditional knock-out mice. **(A)** Representative flow cytometry plots showing gating strategy for CD19^+^ B220^lo^ CD5^+^ CD43^+^ B-1a cells in peritoneal cavity (PC) and blood (left panel). Numbers adjacent to gates indicate cell frequencies in the parent population. Frequencies of B-1a cells in peritoneal cavity and blood (right panel). Bars and error bars represent mean ± SD. **(B)** Natural serum IgM levels. Graph lines and error bars indicate mean ± SD. **(C)** Correlation between serum IgM levels and frequencies of B-1a cells in peritoneum. Data are representative of three independent experiments with 3-13 mice per group for each experiment. **(D)** Anti-NP IgM and IgG3 antibody production in serum from mice 7 days after i.p. immunization with 50 µg NP-Ficoll. Graph lines and error bars indicate mean ± SD. Data are representative of two independent experiments with 3-7 mice per group. **P ≤0.01, ***P ≤0.001, and ****P ≤0.0001.

As IκBNS is required for B-1a cell development as early as during the transitional B-1a cell stage (16), we assessed the B-1a cell compartment in both the peritoneum and blood. We found greatly reduced frequencies of B-1a cells in the *nfkbid* ^B-^ mice compared to *nfkbid* ^B+^ mice (**Figure 3A**). Since B-1 cells are the main producers of natural IgM (23, 24), we evaluated serum IgM levels by ELISA. IgM levels in *nfkbid* ^B-^ mice were partially reduced compared to *nfkbid* ^B+^ mice (**Figure 3B**). In addition, IgM serum levels correlated to the frequencies of B-1a cells detected in the peritoneum (**Figure 3C**). Basal serum levels of IgG2c and IgG3 were also reduced in *nkbid* ^B-^ mice compared to *nfkbid* ^B+^ mice, whereas levels of IgG1, IgG2b, and IgA were normal (**Supplementary Figure 2A**). Absence of IκBNS results in a reduced MZB population, which appears restored upon ageing. However, these accumulating MZB-like cells are phenotypically different compared to the wild type (wt) MZB cells and remain dysfunctional (25). When we analyzed the MZB compartment in the *nfkbid* ^B-^ mice, we observed normal frequencies compared to the control *nfkbid* ^B+^ mice (**Supplementary Figure 2B**). B cells from IκBNS-deficient *bumble* mice also display increased surface IgM level (15), which was not observed in the conditional KO mice B cells (**Supplementary Figure 2C**). To assess the ability of the *nfkbid* ^B-^ mice to respond to TI immunization, we immunized mice i.p. with the TI-2 model antigen NP-Ficoll. Like IκBNS-deficient *bumble* mice, the *nfkbid* ^B-^ mice were unable to produce NP-specific IgM antibodies. The NP-specific IgG3 production in the *nfkbid* ^B-^ mice was also significantly reduced compared to the *nfkbid* ^B+^ control animals (**Figure 3D**). Thus, even though the frequencies of MZBs were normal in the *nfkbid* ^B-^ mice, their ability to respond to TI-2 antigens was impaired.

### IκBNS is dispensable for B cells to respond to T cell-dependent antigens

**Figure 4:**
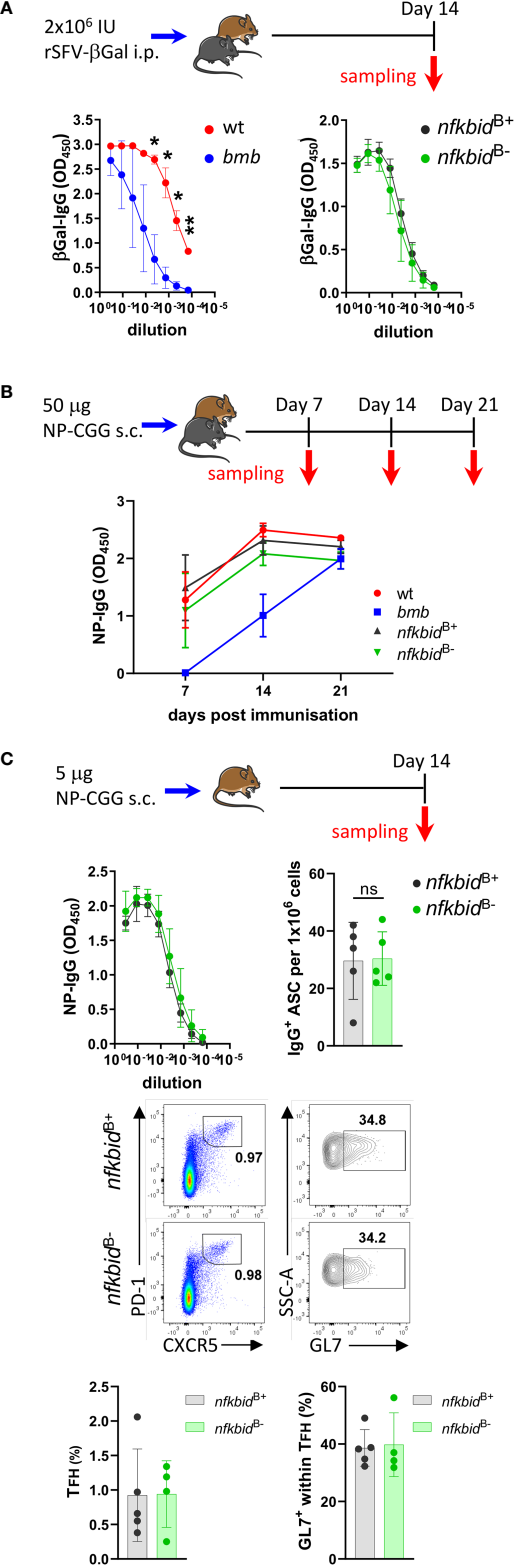
Intact T-dependent response in *nfkbid* ^B-^ conditional knock-out mice. **(A)** Mice were immunized with 2x10^6^ IU rSFV-βGal i.p. and anti-βGalactosidase IgG antibody responses were evaluated in serum from wt, *bumble* (*bmb), nfkbid* ^B+^, and *nfkbid* ^B-^ mice 14 days post immunization. Symbols and error bars indicate mean ± SD. Data are representative of two independent experiments with 3-7 mice per group. **(B)** Mice were immunized with 25 µg NP-CGG in AddaVax s.c. in each flank (i.e. 50 µg NP-CGG in total per mouse), and NP-specific IgG production in serum from wt, *bumble* (*bmb), nfkbid* ^B+^, and *nfkbid* ^B-^ mice evaluated 7, 14, and 21 post immunization. Symbols and error bars represent mean ± SD. Data is from one experiment with 5 mice per group. **(C)** *nfkbid* ^B+^, and *nfkbid* ^B-^ mice were immunized with 2,5 µg NP-CGG in AddaVax s.c. in each flank (i.e. 5 µg NP-CGG in total per mouse), and NP-specific IgG response was evaluated 14 days post immunization. Symbols and error bars represent mean ± SD (upper left panel). NP-specific IgG-producing cells were enumerated using ELISpot assay 14 days post-immunization from splenic B cells. Graph shows frequencies of NP-specific IgG^+^ cells. Mean ± SD are plotted. Data is representative for two independent experiments with 3-5 mice per group (upper right panel). Representative flow cytometry plots showing gating on GL7^+^ CXCR5^+^ PD-1^+^ T_FH_ cells *in vivo* in the immunized *nfkbid* ^B+^ and *nfkbid* ^B-^ mice (middle panels). Frequencies of T_FH_ cells and GL7^+^ T_FH_ cells are shown (lower bar graphs). Bars and error bars indicate mean ± SD. Mouse pictures were created with https://BioRender.com. *P ≤0.05, **P ≤0.01, ns, not significant.

Having established the mouse model and characterized key features of the mice, we turned to the main question of the current study, which was to examine the role of IκBNS in primary antibody responses to TD antigens. To mimic a viral infection, we first immunized wt and *bumble* mice, as well as *nfkbid* ^B+^ and *nfkbid* ^B-^ mice, i.p. with rSFV-βGal, which was used in the ENU screen where the *bumble* mice were originally identified (15). Consistent with previous findings (15), 14 days after immunization the primary βGal-specific IgG production was reduced in *bumble* compared to in wt control mice. In contrast, *nfkbid* ^B-^ mice exhibited similar titers of βGal-specific IgG compared to *nfkbid* ^B+^ mice (**Figure 4A**).

We next used a stronger TD antigen, 50 μg NP-CGG in AddaVax, and evaluated the primary antigen-specific IgG response 7, 14, and 21 days after immunizations. In accordance with previous findings, we observed reduced NP-specific IgG production in the IκBNS-deficient *bumble* mice compared to in wt control mice at day 7. The IgG titers increased in *bumble* between day 7 and 14 but did not reach the levels measured in wt mice (**Figure 4B**). Similarly, generation of GL7^+^ CD95^+^ GC B cells in *bumble* mice was delayed compared to in wt mice (**Supplementary Figure 3**). In contrast, in the *nfkbid* ^B-^ mice the NP-specific IgG production and GC B cell frequencies were comparable to *nfkbid* ^B+^ mice (**Figure 4B** and **Supplementary Figure 3**). As we previously reported, IκBNS is involved in the regulation of antibody producing plasma cell (PC) differentiation (25, 26). Thus, we also evaluated the IgG producing antibody-secreting cell (ASC) generation in NP-CGG/AddaVax-immunized mice 14 days after immunization using lower doses of NP-CGG (5 μg). As for the higher dose of NP-CGG, we observed similar NP-specific IgG production between *nfkbid* ^B-^ and *nfkbid* ^B+^ mice. We also found comparable numbers of IgG-producing ASC by ELISpot analysis (**Figure 4C**, upper panels), suggesting that B cell-intrinsic IκBNS expression was not required for differentiation of IgG-producing ASC following primary immunization with TD antigen. IκBNS has been shown to be required for follicular T helper (T_FH_) cell differentiation during TD antibody responses (19). The frequencies of PD-1^+^ CXCR5^+^ T_FH_ cells and their maturation as evaluated by GL7 expression were comparable between *nfkbid* ^B-^ and *nfkbid* ^B+^ mice (**Figure 4C**, lower panels). These data suggest that the role of IκBNS in B cells during primary B cell responses to the TD immunization is limited, and that the defect in TD responses observed in mice that completely lack IκBNS expression is due to the requirement of IκBNS in other immune cells. Here, we observed normal T_FH_ cell frequencies in NP-CGG/AddaVax immunized *nfkbid* ^B-^ mice, suggesting that their presence was sufficient to restore the TD response.

## Discussion

Whilst the roles of NF-κB transcription factors in B cell development have been studied extensively (27–29), less is known about the specific function of different NF-κB components in B cell function. Studies of mice that lack members of NF-κB signaling pathway demonstrate that reduced or abolished T cell-independent responses is a common characteristic of these deficiencies (28, 30–32). Innate-like B cells, including MZBs and B-1 B cells, which are the main producers of steady-state circulating IgM and are responsible for the prompt response to TI antigens, are present at reduced frequencies in mice with deficient NF-κB signaling (13, 33, 34), as also observed in *bumble* mice (15, 16, 25).

Here, we demonstrate that the absence of IκBNS only in the B cell compartment resulted in severely reduced peritoneal B-1 cell population. Additionally, the conditional KO strain exhibited decreased basal serum IgM levels and impaired antibody responses to TI antigen, which may be explained by the lack of B-1a cells and/or dysfunctional MZBs. Further detailed phenotypic characterization and functional analysis of the MZB population in the conditional KO mice will define their nature and clarify their role in TI immune responses. Further studies will also elucidate why B cells from the *nfkbid* ^B-^ mice displayed comparable surface IgM levels to those in the control *nfkbid* ^B+^ mice, while B cells from *bumble* mice had elevated surface IgM levels compared to the wt control mice.

Previous work showed that GC responses were impaired in mice deficient in *relB* and *nfkb2*, but not in mice deficient in *nfkb1* (35). Impaired GC responses may be due to impaired antigen uptake and processing by innate antigen presenting cells, defective T cell activation or B cell-intrinsic defects affecting B cell activation or differentiation. Mouse strains with B cell-specific deletions of individual NF-κB transcription factors and other components of the NF-κB pathway have been instrumental for studying their function in B cells. Using this strategy, it was found that B cell-intrinsic NEMO expression is required for GC responses (36). The Klein group used GC B cell-specific deletion of the c-Rel and p65 (RelA) subunits and found that GC B cell-specific c-Rel deletion abrogated the formation of GCs, whilst p65 (RelA) deletion did not (32). However, p65 (RelA) deletion did impair generation of GC-derived plasma cells (37).

In the current study, we focused on characterizing the role of IκBNS in primary T cell-dependent antibody responses using the viral antigen, rSFV-βGal, and the widely used hapten-protein model antigen, NP-CGG. Previous studies by Touma et al. found that IκBNS KO mice completely failed to form GCs at five days post administration of sheep red blood cells (17). However, IκBNS-deficient *bumble* mice had normal GC responses 14 days post immunization with NP-CGG (15). The discrepancy between the two prior studies is likely explained by differences in kinetics, since the absence of functional IκBNS delayed but did not ablate the GC response, as demonstrated here. In contrast to in complete IκBNS KO mice, B cell-specific IκBNS deletion did not lead to an impairment in GC formation, suggesting that the delayed GC response in the IκBNS KO mice is due to a B cell-extrinsic defect. In T cells, IL-2 induced proliferation and production of IFNγ and IL-21 has been shown to depend on IκBNS expression (19, 38, 39). Additionally, IκBNS was reported to be directly involved in regulating CXCR5 expression on T_FH_ cells, which enables them to migrate to B cell follicles in secondary lymphoid structures (19). Considering the role of IκBNS in T_FH_ differentiation and function, as well as the importance of IL-21 for plasma cell differentiation in the GC environment, it is possible that the defects in the TD antigen response in IκBNS-deficient mice stem from compromised CD4^+^ T cell help to B cells. Limitations of our study include the fact that quite small number of mice were used in some experiments due to the challenge of maintaining and expanding these strains. For example, this may explain the lack of statistical significance between the experimental and control group in the measurements of serum IgG2c (**Supplementary Figure 2**), which could be revisited in future analyses.

In conclusion, our results suggest that the inability to induce antibody responses to TD antigens in absence of IκBNS does not result from a defect in the B cell compartment but rather from other cellular components involved in the GC reaction. Further studies are required to elucidate how IκBNS directs essential interactions involved in T cell-dependent antibody responses and whether the lack of IκBNS in B cells results in more subtle defects in the antibody response than those investigated here. The findings reported here have implications for our understanding of the NF-κB pathway in regulating adaptive immune responses.

## Data availability statement

The original contributions presented in the study are included in the article/**Supplementary Material**. Further inquiries can be directed to the corresponding author.

## Ethics statement

The animal study was reviewed and approved by Committee for Animal Ethics (Stockholms Norra Djurförsöksetiska nämnd).

## Author contributions

SK and MÁ designed the study, carried out experiments, interpreted and analysed data and wrote the manuscript. DSS and JS performed experiments, and analysed data. JR and BB designed research and provided scientific input. GKP interpreted data and provided scientific input. GKH provided resources for experiments, designed the study, provided scientific input, and wrote the manuscript. All authors commented and edited the manuscript. All authors contributed to the article and approved the submitted version.

## Funding

This work was supported by a Karolinska Institutet Doctoral grant to SK, a research grant from the Swedish Research Council to GKH (agreement 2017-00968) and an equipment grant from the Fondation Dormeur Vaduz to GKH.

## Acknowledgments

We thank the personnel at the animal facilities at the Department of Microbiology, Tumor and Cell Biology and Comparative Medicine at Karolinska Institutet for expert assistance. We also thank Dr Michael Reth and Dr Stephen Malin for making the *Cd79a* ^Cre^ mice available to us. We are grateful to Gerald McInerney for providing rSFV-βGal, and to Xaquin Castro Dopico, Remy Muts, Sanjana Narang and Nikolaos Pantouloufos for technical assistance.

## Conflict of interest

The authors declare that the research was conducted in the absence of any commercial or financial relationships that could be construed as a potential conflict of interest.

## Publisher’s note

All claims expressed in this article are solely those of the authors and do not necessarily represent those of their affiliated organizations, or those of the publisher, the editors and the reviewers. Any product that may be evaluated in this article, or claim that may be made by its manufacturer, is not guaranteed or endorsed by the publisher.
