# Supplementary material for: IκBNS expression in B cells is dispensable for IgG responses to T cell-dependent antigens
Source: Front Immunol. 2022 Oct 21;13:1000755. doi: 10.3389/fimmu.2022.1000755 (PMC9634154; doi:10.3389/fimmu.2022.1000755)
Supplement: Supplementary file 1 [file Presentation_1.pptx]

## Slide 1
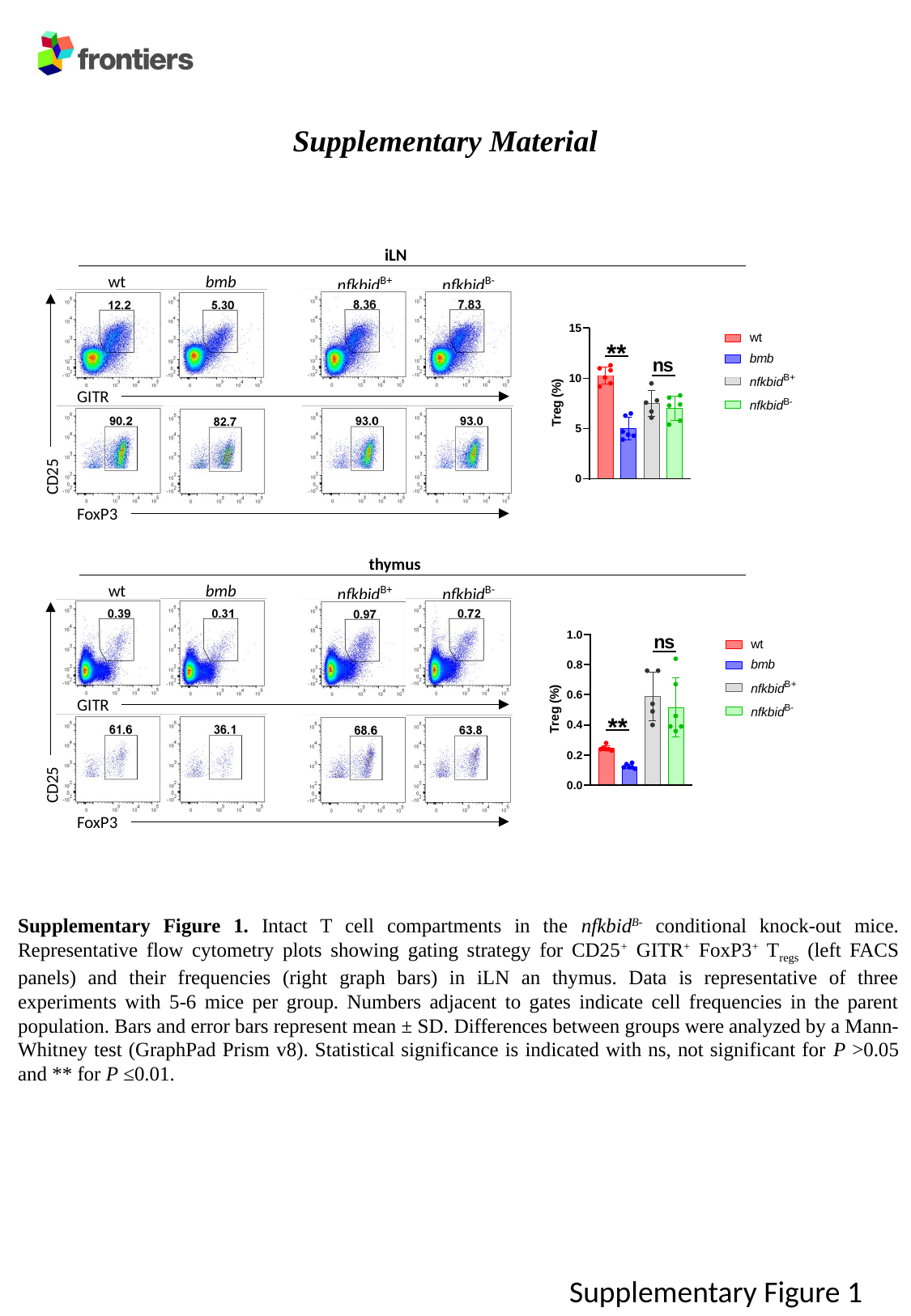

Supplementary Material
iLN
wt
bmb
nfkbidB+
nfkbidB-
GITR
CD25
FoxP3
thymus
wt
bmb
nfkbidB+
nfkbidB-
GITR
CD25
FoxP3
Supplementary Figure 1. Intact T cell compartments in the nfkbidB- conditional knock-out mice. Representative flow cytometry plots showing gating strategy for CD25+ GITR+ FoxP3+ Tregs (left FACS panels) and their frequencies (right graph bars) in iLN an thymus. Data is representative of three experiments with 5-6 mice per group. Numbers adjacent to gates indicate cell frequencies in the parent population. Bars and error bars represent mean ± SD. Differences between groups were analyzed by a Mann-Whitney test (GraphPad Prism v8). Statistical significance is indicated with ns, not significant for P >0.05 and ** for P ≤0.01.
Supplementary Figure 1

## Slide 2
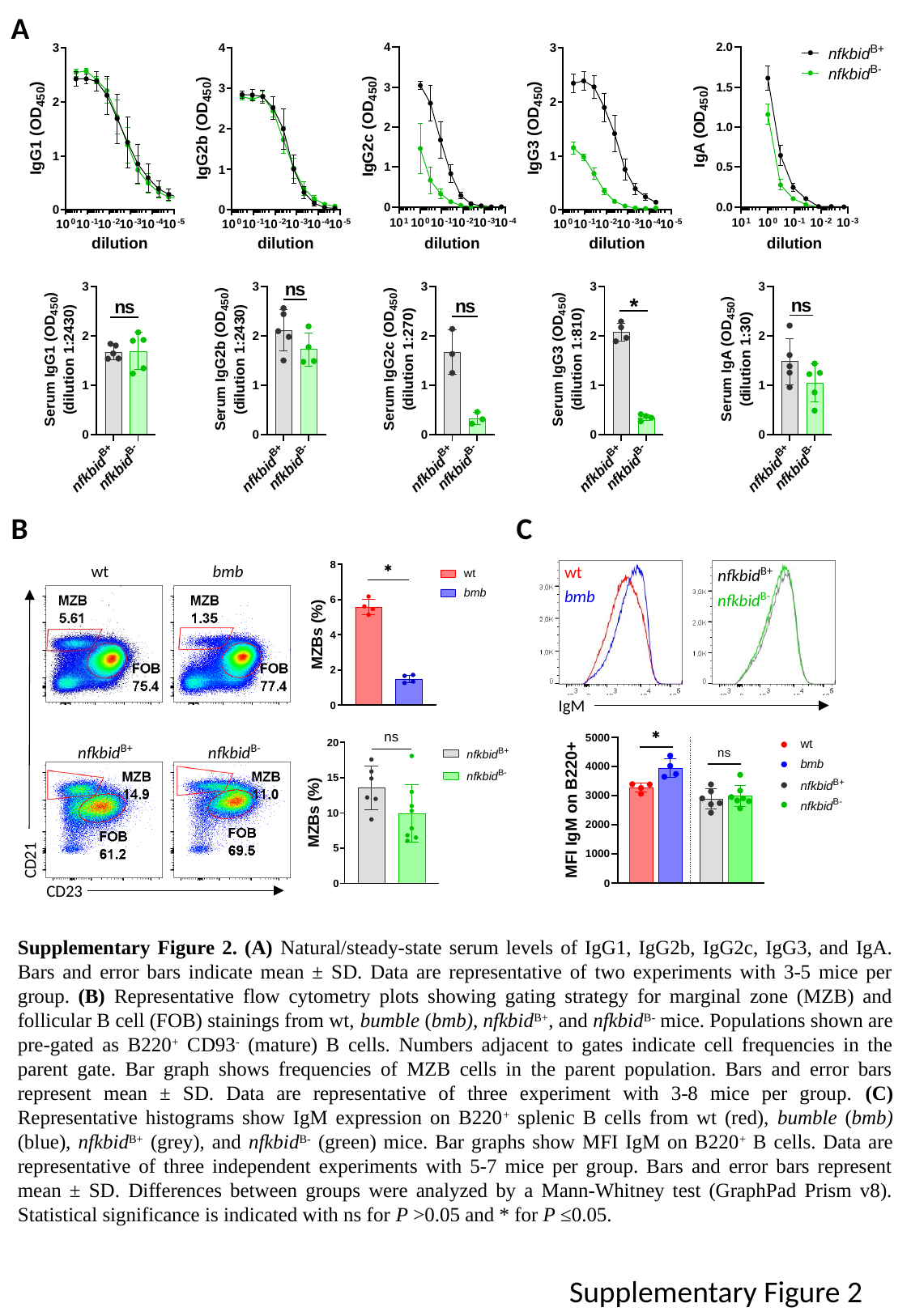

A
B
wt
bmb
nfkbidB+
nfkbidB-
CD21
CD23
C
wt
nfkbidB+
bmb
nfkbidB-
IgM
Supplementary Figure 2. (A) Natural/steady-state serum levels of IgG1, IgG2b, IgG2c, IgG3, and IgA. Bars and error bars indicate mean ± SD. Data are representative of two experiments with 3-5 mice per group. (B) Representative flow cytometry plots showing gating strategy for marginal zone (MZB) and follicular B cell (FOB) stainings from wt, bumble (bmb), nfkbidB+, and nfkbidB- mice. Populations shown are pre-gated as B220+ CD93- (mature) B cells. Numbers adjacent to gates indicate cell frequencies in the parent gate. Bar graph shows frequencies of MZB cells in the parent population. Bars and error bars represent mean ± SD. Data are representative of three experiment with 3-8 mice per group. (C) Representative histograms show IgM expression on B220+ splenic B cells from wt (red), bumble (bmb) (blue), nfkbidB+ (grey), and nfkbidB- (green) mice. Bar graphs show MFI IgM on B220+ B cells. Data are representative of three independent experiments with 5-7 mice per group. Bars and error bars represent mean ± SD. Differences between groups were analyzed by a Mann-Whitney test (GraphPad Prism v8). Statistical significance is indicated with ns for P >0.05 and * for P ≤0.05.
Supplementary Figure 2

## Slide 3
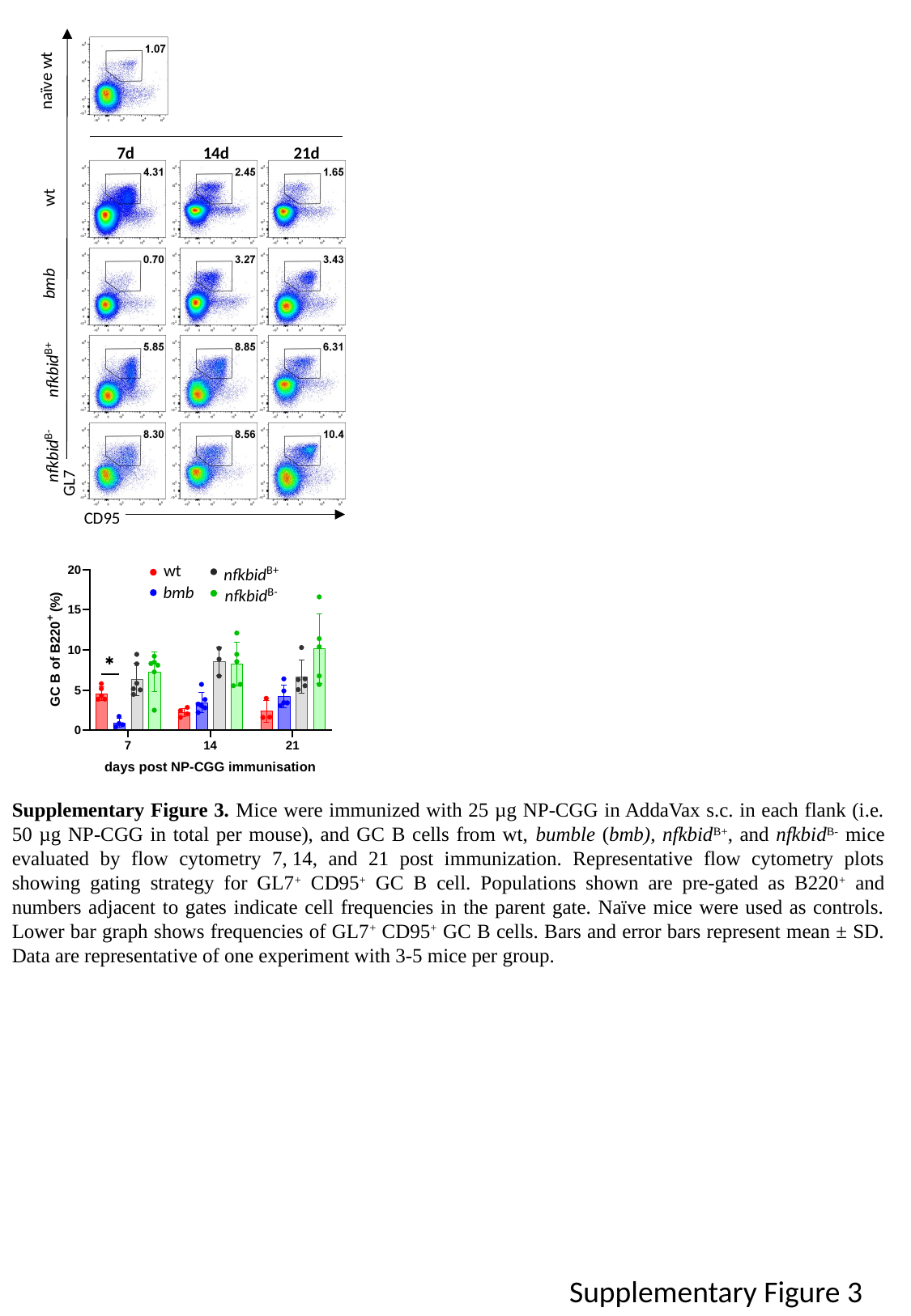

naïve wt
7d
14d
21d
wt
bmb
nfkbidB+
nfkbidB-
GL7
CD95
wt
nfkbidB+
bmb
nfkbidB-
Supplementary Figure 3. Mice were immunized with 25 µg NP-CGG in AddaVax s.c. in each flank (i.e. 50 µg NP-CGG in total per mouse), and GC B cells from wt, bumble (bmb), nfkbidB+, and nfkbidB- mice evaluated by flow cytometry 7, 14, and 21 post immunization. Representative flow cytometry plots showing gating strategy for GL7+ CD95+ GC B cell. Populations shown are pre-gated as B220+ and numbers adjacent to gates indicate cell frequencies in the parent gate. Naïve mice were used as controls. Lower bar graph shows frequencies of GL7+ CD95+ GC B cells. Bars and error bars represent mean ± SD. Data are representative of one experiment with 3-5 mice per group.
Supplementary Figure 3

## Slide 4
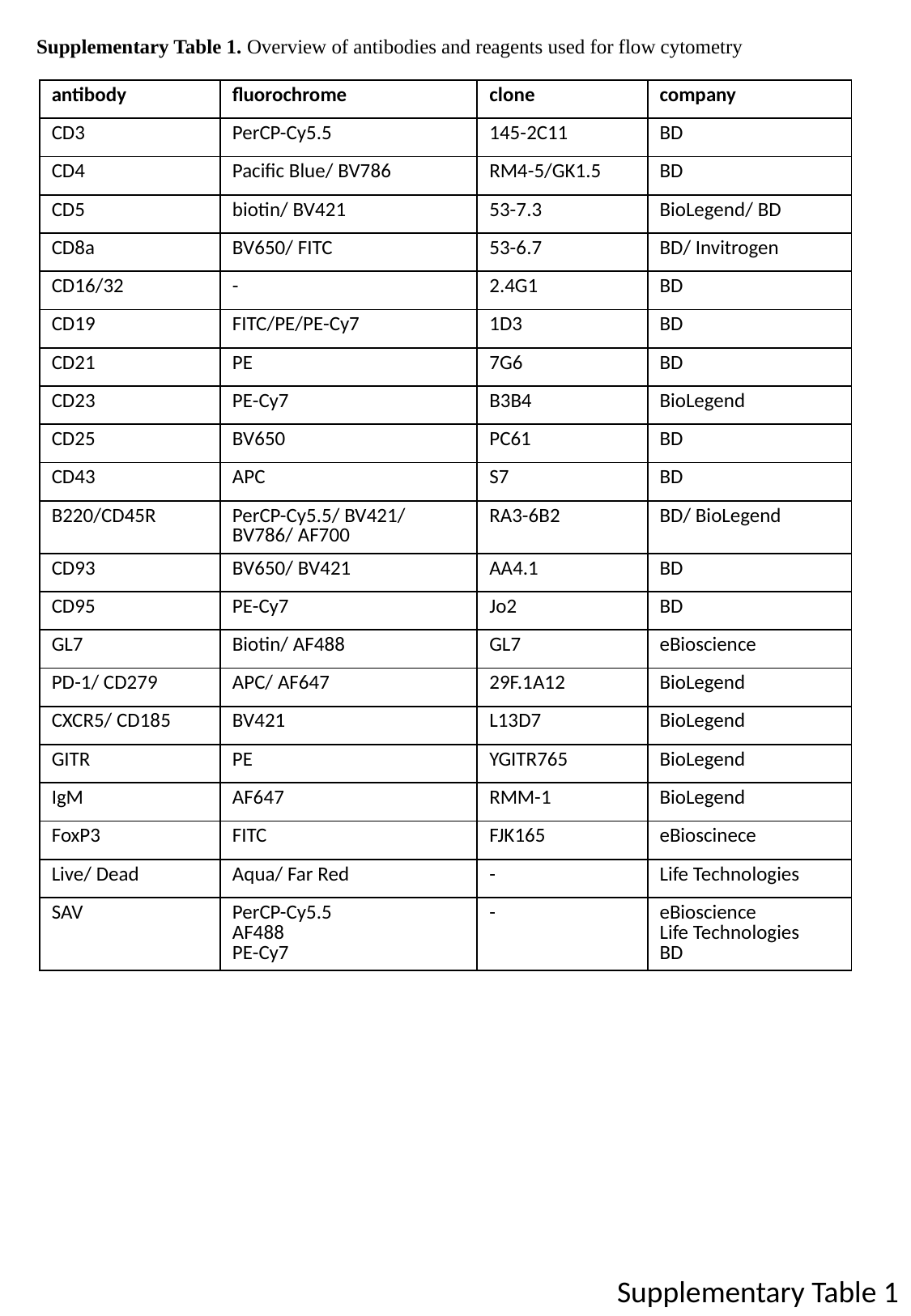

Supplementary Table 1. Overview of antibodies and reagents used for flow cytometry
| antibody | fluorochrome | clone | company |
| --- | --- | --- | --- |
| CD3 | PerCP-Cy5.5 | 145-2C11 | BD |
| CD4 | Pacific Blue/ BV786 | RM4-5/GK1.5 | BD |
| CD5 | biotin/ BV421 | 53-7.3 | BioLegend/ BD |
| CD8a | BV650/ FITC | 53-6.7 | BD/ Invitrogen |
| CD16/32 | - | 2.4G1 | BD |
| CD19 | FITC/PE/PE-Cy7 | 1D3 | BD |
| CD21 | PE | 7G6 | BD |
| CD23 | PE-Cy7 | B3B4 | BioLegend |
| CD25 | BV650 | PC61 | BD |
| CD43 | APC | S7 | BD |
| B220/CD45R | PerCP-Cy5.5/ BV421/ BV786/ AF700 | RA3-6B2 | BD/ BioLegend |
| CD93 | BV650/ BV421 | AA4.1 | BD |
| CD95 | PE-Cy7 | Jo2 | BD |
| GL7 | Biotin/ AF488 | GL7 | eBioscience |
| PD-1/ CD279 | APC/ AF647 | 29F.1A12 | BioLegend |
| CXCR5/ CD185 | BV421 | L13D7 | BioLegend |
| GITR | PE | YGITR765 | BioLegend |
| IgM | AF647 | RMM-1 | BioLegend |
| FoxP3 | FITC | FJK165 | eBioscinece |
| Live/ Dead | Aqua/ Far Red | - | Life Technologies |
| SAV | PerCP-Cy5.5 AF488 PE-Cy7 | - | eBioscience Life Technologies BD |
Supplementary Table 1
